# Maximal Cytoreductive Effort in the Time of Pandemic: Outcomes for Patients with Advanced Ovarian Cancer and Lessons for the Future

**DOI:** 10.3390/cancers18152403

**Published:** 2026-07-25

**Authors:** Porfyrios Korompelis, Anke Smits, Ioanna Antoniou, Baljinder Mann, Callum Betts, Viktor Cassar, V. B. Swetha Rongali, Christine Ang, Dominic Blake, Stuart Rundle

**Affiliations:** 1 NGOC, Gateshead NHS Foundation Trust, Gateshead NE9 6SX, UK; 2 BHGOC, Barts Health NHS Trust, London E1 1FR, UK; 3 Newcastle Hospitals NHS Foundation Trust, Newcastle upon Tyne NE1 4LP, UK; 4 Country Durham & Darlington NHS Foundation Trust, Darlington DL3 6HX, UK; 5 Medical School, Newcastle University, Newcastle upon Tyne NE1 7RU, UK; 6 Mater Dei Hospital, MSD 2090 Msida, Malta; 7 Sandwell & West Birmingham NHS Trust, West Bromwich B71 4HJ, UK

**Keywords:** advanced ovarian cancer, maximal-effort cytoreductive surgery, COVID-19 pandemic, survival, oncological outcome

## Abstract

***Background***: The COVID-19 pandemic substantially disrupted healthcare systems worldwide, raising concerns regarding delays in diagnosis, alterations in treatment pathways, and oncological outcomes for women with advanced ovarian cancer (AOC). This study evaluated the impact of the pandemic on the management and survival of patients with AOC treated at a high-volume ESGO-accredited tertiary gynaecological oncology centre. ***Methods*****:** A retrospective cohort study was conducted using a prospectively maintained database of consecutive women diagnosed with FIGO stage III–IV epithelial ovarian, fallopian tube or primary peritoneal cancer between January 2017 and December 2022. Patients were divided into pre-pandemic and pandemic cohorts. Demographic characteristics, FIGO stage, treatment pathways, surgical outcomes, and overall survival were compared. Survival was analysed using Kaplan–Meier methodology and multivariable Cox proportional hazards regression was performed to evaluate the independent association between the pandemic period and overall survival after adjustment for age, FIGO stage, ECOG performance status and treatment modality. ***Results*****:** A total of 700 patients were included, 406 patients treated before the pandemic and 294 during the pandemic. Patients diagnosed during the pandemic had a better ECOG performance status, but 51.3% presented with FIGO stage IV disease. Overall treatment rates were maintained (84.4% vs. 87.4% pre-pandemic), although treatment strategies shifted towards increased use of neoadjuvant chemotherapy (55% vs. 42.4%, *p* < 001), with a greater proportion of patients subsequently undergoing interval debulking surgery. Complete cytoreduction following interval debulking surgery was preserved throughout the study period, despite more advanced disease at presentation. One-year overall survival was similar between cohorts (69% vs. 72.2% pre-pandemic), whereas three-year overall survival was significantly lower among patients treated during the pandemic (36.1% vs. 45.1%). On multivariable Cox regression analysis, treatment during the pandemic remained independently associated with poorer overall survival after adjustment for clinically relevant baseline factors. ***Conclusions*****:** Despite unprecedented disruption to healthcare services, specialist gynaecological oncology care has maintained access to multimodality treatment and preserved maximal effort cytoreductive surgery. Nevertheless, patients diagnosed during the pandemic experienced inferior long-term survival independent of baseline disease characteristics and treatment allocation, highlighting the broader indirect consequences of the pandemic on cancer outcomes. These findings emphasise the importance of protecting diagnostic pathways, multidisciplinary cancer services and surgical capacity during future healthcare crises.

## Simple Summary

The COVID-19 pandemic profoundly disrupted healthcare delivery and raised concerns regarding its impact on the management and outcomes of women with advanced ovarian cancer (AOC). We conducted a retrospective cohort study of 700 consecutive patients with FIGO stage III–IV ovarian cancer treated at an ESGO-accredited tertiary gynaecological oncology centre between January 2017 and December 2022. Patients diagnosed before and during the pandemic were compared with respect to clinicopathological characteristics, treatment pathways, surgical outcomes and overall survival. Survival was assessed using Kaplan–Meier analysis and multivariable Cox proportional hazards regression. Patients diagnosed during the pandemic had a better ECOG performance status but presented more frequently with FIGO stage IV disease. Overall access to treatment was maintained, although management shifted towards greater use of neoadjuvant chemotherapy, with a higher proportion of patients subsequently undergoing interval debulking surgery. Complete cytoreduction following interval debulking surgery was preserved despite the higher burden of advanced disease. Although one-year overall survival was similar between cohorts, three-year survival was significantly poorer among patients treated during the pandemic, and treatment during the pandemic remained independently associated with inferior overall survival after adjustment for age, FIGO stage, ECOG performance status and treatment modality. These findings demonstrate that specialist gynaecological oncology services were able to preserve multimodality treatment pathways and maximal effort cytoreductive surgery despite unprecedented healthcare disruption. However, the persistence of poorer long-term survival following multivariable adjustment highlights the broader indirect consequences of the pandemic on cancer outcomes and emphasises the importance of maintaining timely diagnosis, referral pathways and specialist cancer services during future healthcare emergencies.

## 1. Introduction

The COVID-19 pandemic has resulted in a worldwide toll of approximately 21 million cases, causing more than 190,000 deaths in the three years to March 2023 [1]. Whilst the numbers of direct COVID-19 deaths are relatively easy to estimate in resource-rich health settings, estimating the magnitude of the impact of excess deaths due to poorer diagnosis and management of specific cancers is more challenging. Significant disruption to health systems was necessary in order to help prevent COVID-19 spread, to safeguard critical health infrastructure and because of the overwhelming pressure the pandemic put on medical facilities across the globe. These pressures had a multitude of effects on many systems’ capacity for screening, diagnosis and timely treatment for cancer patients [2,3]. At the peak of the pandemic in the USA, diagnostic rates for malignancies including breast, prostate, colon, and lung cancers were reduced by up to 85% [4]. Furthermore, over one-third of all elective cancer surgeries are estimated to have been cancelled or postponed in the first 12 weeks after the declaration of an international public health emergency by the World Health Organisation in March 2019 [5].

Ovarian cancer patients are usually diagnosed at an advanced stage, and this disease is unusual amongst other solid organ cancers. Widely disseminated and advanced-stage disease is treated primarily with an approach aimed at reducing tumour burden to no detectable macroscopic tumour through a combination of cytotoxic chemotherapy and aggressive surgical cytoreduction of abdominal-pelvic peritoneal disease [6,7]. Due to the nature of the surgery necessary for completion of the gold standard treatment and the effects of the pandemic on healthcare systems, OC patients were extremely vulnerable to disruption of their treatment, including delays or cancellations to surgery. In the UK, 54% of women diagnosed with ovarian cancer reported that their treatment had been affected by the COVID-19 pandemic, and 27% said that they were not able to access the same care and support as before the pandemic [8].

While several studies have reported alterations in treatment pathways during the pandemic, there is limited evidence regarding whether these changes translated into differences in long-term oncological outcomes after adjustment for baseline disease characteristics and treatment allocation [9,10]. The primary aim of our study is to evaluate the impact of the pandemic on the management and outcomes of advanced ovarian cancer in a high-volume ESGO-accredited centre in the UK. Secondary aims are to assess differences in treatment pathways and overall survival, including adjusted survival outcomes in 1 and 3 years for patients treated during and after the immediate pandemic. The results may give us valuable information with which we can tailor ovarian cancer patients’ treatment outside the pandemic. In addition, they may help us prepare for similar unexpected health emergencies in the future.

## 2. Methodology

All patients with biopsy-proven advanced ovarian cancer (AOC), of International Federation of Gynaecology and Obstetrics (FIGO) stage IIIC and stage IV who were referred to the Northern Gynaecological Oncology Centre (NGOC), Queen Elizabeth Hospital, Gateshead from March 2017 to March 2022 were identified from the prospectively maintained departmental patient database. Patient demographics and clinico-pathological data relating to comorbidities, Eastern Cooperative Oncology Group (ECOG) performance status (PS), treatment and outcomes were extracted from patients’ electronic and hospital-based case records. Detailed follow-up data relating to survival and recurrence were collected and cross-referenced with primary care records and the death register.

Cases were divided into two groups based on date of treatment. The COVID (study) group was treated in the period March 2020 to March 2022. This period was selected to commence coincidentally with the declaration of the pandemic and the initiation of legal restrictions on movement and social gatherings in the UK, and to end with the final abolition of these restrictions two years later [11]. The comparator group underwent treatment in the three years prior to the pandemic in the same high-volume NGOC (March 2017–February 2020).

Standard treatment protocols for the management of advanced ovarian cancer were applied according to the prevailing local health system pressures and national treatment recommendations during the pandemic. Where surgery was undertaken as part of the treatment of advanced ovarian cancer, this was with the intent of maximal effort for complete resection of macroscopic disease in all cases.

Data were analysed using IBM SPSS V.30 Statistics. Continuous variables were assessed for distribution and are presented as median and range, or median and interquartile range, as appropriate. Categorical variables are presented as frequencies and percentages. Comparisons between the pre-pandemic and pandemic cohorts were performed using the Mann–Whitney U test for non-normally distributed continuous variables and the Chi-squared test or Fisher’s exact test for categorical variables, as appropriate. Overall survival was calculated from the date of MDT discussion/referral to the NGOC until death from any cause or last known follow-up. Patients who were alive at last follow-up were censored. Survival data were updated for both cohorts to the end of June 2026. Kaplan–Meier survival curves were generated to estimate overall survival, and survival distributions were compared between groups using the log-rank test. Cox proportional hazards regression analysis was used to evaluate the association between treatment period and overall survival. Where appropriate, multivariable Cox regression was performed to adjust for clinically relevant baseline covariates, including age, FIGO stage, ECOG performance status and treatment modality. Results are presented as hazard ratios with 95% confidence intervals. A two-sided *p*-value of <0.05 was considered statistically significant.

## 3. Results

### 3.1. Patient and Disease Characteristics at Presentation

A total of 700 patients are included in the analysis. During the study period, March 2020 to March 2022, 294 new patients with AOC were referred to the NGOC multi-disciplinary team meeting (MDTM) for treatment planning. This compared with 406 similar patients referred in the preceding three years. The median age at presentation was 67 years (range 27–90 years) in the study group and 69 years (range 21–91 years) in the comparator group. The groups were similarly balanced in the distribution of histological subtypes represented, and FIGO stage 3C disease was the most prevalent stage at diagnosis across both time periods. FIGO stage IV disease at presentation was more prevalent within the study group compared to the comparator group (69.7% vs. 48.6%, *p* < 0.01). Patients who presented before the pandemic had a poorer performance status (PS 2 or more), compared to the pandemic period (47.0% versus 19.7%, *p* < 0.001). Details relating to the baseline demographic and clinico-pathologic characteristics of the groups are given in Table 1.

### 3.2. Treatment of AOC

Of all patients referred with AOC, 84.4% received a form of treatment (i.e., chemotherapy, surgery or a combination) during the pandemic, compared to 87.4% during the pre-pandemic period (*p* = 0.244). Detailed comparison regarding the treatment modalities and outcomes is shown in Table 2.

The pandemic prompted a significant shift towards neo-adjuvant chemotherapy (NACT) as the most prevalent initial treatment. Fifty-five per cent of patients who initiated treatment did so with chemotherapy during the pandemic compared with 42.4% in the pre-pandemic group (*p* < 0.01).

The proportion of all patients undergoing any cytoreductive surgery in either the primary (up-front) or interval (after NACT) setting was similar in pandemic vs. pre-pandemic settings at 63.3% vs. 61.9%, respectively. The complete cytoreduction rate was higher in the pre-COVID group for primary surgery (*p* = 0.014), but did not differ at interval setting (*p* = 0.897).

In the patients who underwent up-front surgery pre-pandemic, 95.1% received adjuvant platinum-based chemotherapy, and 91.9% of patients during the pandemic (*p* = 0.407). Of the patients treated with NACT as the first anti-cancer treatment, significantly more patients had the opportunity for interval cytoreductive surgery during the pandemic (59.3% vs. 41.9%, *p* = 0.001).

Looking at the referral pathway for these patients, the timings of the appointments and the surgical treatment, we did not identify any changes between the COVID and pre-COVID period. In both periods, all patients were seen by a member of the surgical team within 2 weeks of the MDT discussion, and the vast majority of them had their surgical treatment within 4 weeks of the appointment, before and during the pandemic.

**Figure 1:**
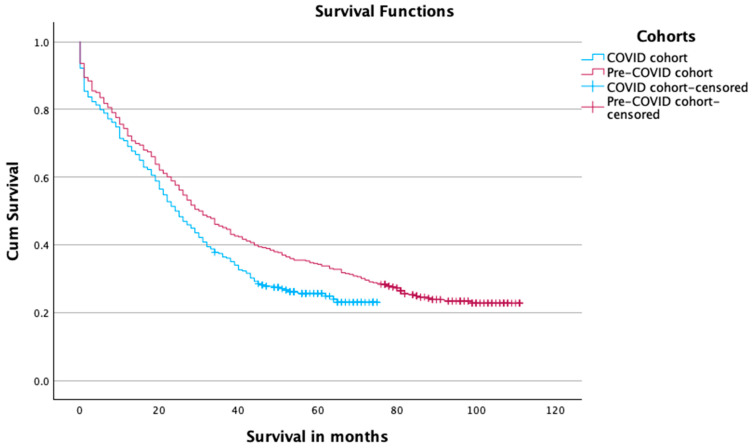
Overall survival.

Overall, the 1-year survival for the patients who received treatment during the pandemic and pre-pandemic was similar, with 69.0% versus 72.2% *p* = 0.370). However, the 3-year survival was significantly lower in the pandemic cohort (36.1%) compared to the historical cohort (45.1%) (*p* = 0.017). In addition, we illustrated the survival of the two groups in the Kaplan–Meijer curve, which was significantly poorer in the COVID group (*p* = 0.012) (Figure 1). After adjustment for age at diagnosis, ECOG performance status, FIGO stage and treatment modality, patients in the COVID cohort had a 39% higher hazard of death compared with patients treated in the pre-COVID period (HR 1.39, 95% CI 1.14–1.69, *p* < 0.001).

## 4. Discussion and Lessons to Be Learnt

The COVID-19 pandemic led to an unprecedented shock to all aspects of our lives, and the primary focus of the health systems shifted to the management of COVID-19. As a result, cancer patients have witnessed remarkable challenges including cancellation of face-to-face clinical reviews, late referrals to tertiary services, delays in laboratory test results, imaging studies and treatments [11]. In our paper, we investigate possible changes to practice in the management of advanced ovarian cancer during the pandemic and the outcomes for this cohort of patients.

According to our data, there is no difference in the age of presentation or in the distribution of the different histological subtypes. On the other hand, there was a statistically significant increase in FIGO stage IV disease at presentation during the pandemic (69.7% vs. 48.6%, *p* < 0.01). The reason behind this reality is not profound. The obvious interpretation sits with the later presentation and diagnosis during the lockdown, reflecting the detrimental effect of COVID-19 in the management of patients with advanced OC. In addition, our team was part of the MROC trial from 2017 to 2019, and this involvement may have changed the way CT findings are reported, contextualised, and integrated into surgical decision-making.

Additionally, patients who presented during the pandemic had better performance status. Patients before the pandemic had a poorer performance status (PS 2 or more), compared to the pandemic period (47.0% versus 19.7%, *p* < 0.001). This is a paradox, given the fact that half of them presented with stage IV disease, which naturally comes with an increased tumour burden and clinical deterioration. A possible interpretation is that during the lockdown, patients with preserved functional status had better access to primary care, whereas frailer patients may have been less likely to visit their GPs and undergo tertiary referral.

In our cohort, 84.4% of patients received a form of treatment during the pandemic compared with 87.4% before the pandemic (*p* = 0.244). These findings suggest that overall access to treatment was largely maintained despite substantial pressures across the healthcare system. Notably, treatment rates during the pandemic remained comparable to or higher than those reported in the National Ovarian Cancer Audit Feasibility Pilot, in which 73.8% of women with stage II–IV ovarian cancer accessed cancer treatment [12].

The pandemic also prompted a significant shift towards neo-adjuvant chemotherapy as the most prevalent initial treatment (55.1% vs. 42.4% pre-COVID, *p* < 0.01). The ovarian cancer surgical service at the NGOC has a reputation for high-volume primary cytoreductive surgery for advanced ovarian cancer, with historically more patients undergoing primary surgery in preference to NACT than many other centres in the UK. The stage shift towards a higher prevalence of stage 4 disease within the study period, as well as the need to carefully consider both timing and anticipated surgical radicality, resulted in a shift towards a greater utilisation of NACT. This shift was necessary to balance risk for patients that may result from longer hospital stays or greater short-term operative morbidity associated with primary surgery [13], and to minimise resultant pressures on scarce elective critical care resources. Greater utilisation of NACT is in keeping with prevailing guidance issued by national and international gynaecologic oncology societies [14]. Importantly, the increased use of NACT did not reduce the proportion of patients proceeding to interval debulking surgery. On the contrary, a significantly greater proportion underwent interval cytoreduction during the pandemic, suggesting that careful patient selection and preservation of surgical capacity allowed completion of multimodal treatment despite service pressures.

Despite the shift towards NACT, the maximal-effort cytoreductive surgery remained a central component of management throughout the pandemic period, demonstrating that it was possible to maintain a high-quality radical surgical service for patients with advanced ovarian cancer throughout the COVID-19 pandemic. In our cohort, the proportion of all patients undergoing any cytoreductive surgery in either the primary (up-front) or interval (after NACT) setting was similar in pandemic vs. pre-pandemic settings at 63.3% vs. 61.9%, respectively. In addition, the complete cytoreduction rate was higher in the pre-COVID group for primary surgery (*p* = 0.014), but did not differ at interval setting (*p* = 0.897). Although complete cytoreduction following primary surgery was lower during the pandemic, interval cytoreductive surgery achieved equivalent rates of complete resection. This finding suggests that increased utilisation of NACT may have partially mitigated the effect of more advanced disease at presentation. Evidence from other providers indicates that maintenance of the surgical offering was not universal and that many patients with proven advanced ovarian cancer were not able to access gold standard treatment [2].

During our project, we investigated any possible delays in the referral pathways, focused on the timing between the MDT discussion and the 1st appointment with the surgical team and the period between the 1st appointment and the surgical treatment, but we did not identify any changes comparing the COVID to the pre-COVID period. In both periods, all patients have been offered an appointment to see a member of the surgical team within 2 weeks from the MDT discussion, and the vast majority of them had their surgical treatment within 4 weeks from the appointment, following the NHS England guidance for cancer waiting times [15,16,17]. These results demonstrate that it was possible to maintain a high-quality radical surgical service for patients with advanced ovarian cancer throughout the COVID-19 pandemic, despite the evidence from other providers indicating that maintenance of the surgical offering was not universal and that many patients with proven advanced ovarian cancer were not able to access gold standard treatment [2,18]. The conditions that allowed for the continued surgical service were met through a combined effort and will of our health system to support cancer surgery, as well as our particular situation of being a sole tertiary referral cancer surgical service in a district general hospital setting, without competing specialities requiring similar high-volume cancer surgery delivery.

We finally evaluated the difference in the survival of these patients, 1 and 3 years after original diagnosis. In the UK, the 5-year survival is 30% for FIGO Stage III ovarian cancer and 15% for stage IV disease, with the overall survival further reduced in the most deprived population, including the NGOC’s catchment area. In our cohort, the 1-year survival for the patients who received treatment during the pandemic and pre-pandemic was similar, with 69.0% versus 72.2% *p* = 0.370). However, the 3-year survival was significantly lower in the pandemic cohort (36.1%) compared to the historical cohort (45.1%) (*p* = 0.017). After adjustment for age at diagnosis, ECOG performance status, FIGO stage and treatment modality, patients in the COVID cohort had a 39% higher hazard of death compared with patients treated in the pre-COVID period (HR 1.39, 95% CI 1.14–1.69, *p* < 0.001). These findings are clinically important but should be interpreted cautiously. The persistence of inferior survival despite adjustment for baseline characteristics suggests that factors beyond stage and treatment selection contributed to poorer outcomes. These may include delays before referral, reduced access to supportive care, interruptions in systemic therapy, COVID-19 infection itself, or broader health-system pressures that cannot be fully captured within retrospective datasets. This observation highlights the complex indirect consequences of the pandemic on cancer outcomes beyond immediate treatment delivery.

Our study has several important strengths. It represents one of the largest single-centre contemporary series evaluating the impact of the COVID-19 pandemic on the management of advanced ovarian cancer within an ESGO-accredited centre. All patients were identified from a prospectively maintained database, treatment pathways were evaluated in detail, and survival outcomes were analysed using Kaplan–Meier methodology and multivariable Cox regression to account for important baseline clinical differences between cohorts. In addition, these findings have important implications for future healthcare emergencies, suggesting that preserving surgical capacity alone may be insufficient; timely diagnosis, referral pathways, systemic therapy delivery and supportive oncology services must also remain protected to minimise long-term adverse oncological outcomes.

Our study also has its limitations. Its retrospective observational design introduces the possibility of residual confounding despite multivariable adjustment. The study reflects the experience of a single high-volume tertiary referral centre, which may limit generalisability to other healthcare settings. In addition, information regarding COVID-19 infection status is not available and therefore could not be incorporated into the adjusted analyses.

Historians say that pandemics end when either they are medically resolved, or fear about the disease wanes. Although patients treated during the pandemic experienced inferior long-term survival, the maintenance of overall treatment rates, preservation of surgical pathways and continued delivery of maximal effort cytoreduction demonstrate the resilience of specialist gynaecological oncology services during an unprecedented healthcare crisis. To avoid future disruptions within the surgical oncology setting, an improved operational infrastructure alongside the necessary supporting oncology staff—without any redeployment of theatre staff away from essential surgical oncology care and time-critical surgery—will be critical to maintaining scheduled surgical oncology activity. The pandemic revealed the vulnerability of our health system but also gave us a unique opportunity for knowledge generation and innovation in delivering the best possible care for our patients. Embracing new technology, efficient workflow of screening, triage, diagnosis and treatment will ensure the quality of healthcare access to patients suffering from malignancies. But above and beyond, it valued the communication and the shared decision-making between cancer patients and their treating clinicians, especially at times of great uncertainty.

## 5. Conclusions

The COVID-19 pandemic significantly influenced the presentation and management of advanced ovarian cancer. Although patients presented with more advanced disease, overall access to treatment was maintained through adaptation of treatment pathways, including greater utilisation of neoadjuvant chemotherapy while preserving maximal-effort cytoreductive surgery within a specialist gynaecological oncology centre. Despite these service adaptations, patients diagnosed during the pandemic experienced inferior long-term overall survival that remained independently associated with the pandemic period after adjustment for important clinical and treatment-related factors. These findings suggest that maintaining surgical capacity is feasible but alone is insufficient to mitigate the indirect effects of healthcare disruption during the pandemic. Protecting timely diagnosis, referral pathways, multidisciplinary decision-making, systemic therapy delivery and specialist surgical services should remain priorities in future healthcare emergencies to minimise adverse oncological outcomes.

## Figures and Tables

**Table 1:** Patient demographics and clinical characteristics.

			Pre-COVID (March 2017–March 2020) *N* = 406	During COVID (March 2020–March 2022) *N* = 294	
Number of patients per year			135	147	
Median age (range)			69 (21–91)	67 (27–90)	0.580
ECOG Performance status					
	0		70 (17.2%)	91 (31.0%)	*p* < 0.001
	1		144 (35.5%)	141 (48.0%)	
	2		121 (29.8%)	42 (14.3%)	
	3		65 (16.0%)	14 (4.8%)	
	4		5 (1.2%)	2 (0.7%)	
	Unknown		1 (0.2%)	4 (1.4%)	
FIGO stage					*p* < 0.001
	Stage III				
		IIIA	35 (8.6%)	10 (3.4%)	
		IIIB	26 (6.4%)	13 (4.4%)	
		IIIC	222 (54.7%)	120 (40.8%)	
	Stage IV				
		IV A	51 (12.6%)	36 (12.2%)	
		IV B	72 (17.7%)	115 (39.1%)	
Histology					*p* = 0.217
	HGSC		320 (78.8%)	246 (83.7%)	
	LGSC		22 (5.4%)	11 (3.7%)	
	Clear Cell		12 (3.0%)	7 (2.4%)	
	Endometrioid		11 (2.7%)	2 (0.7%)	
	Mixed & others		28 (6.9%)	24 (8.2%)	
	Unknown		13 (3.2%)	4 (1.4%)	

**Table 2:** Treatment modalities and survival outcomes.

		Pre-COVID (March 2017–March 2020) *N* = 406	During COVID (March 2020–March 2022) *N* = 294	
Any form of treatment		355 (87.4%)	248 (84.4%)	*p* = 0.244
Type of treatment				*p* < 0.01
	Primary CR	185 (45.1%)	86 (29.3%)	
	NACT	172 (42.4%)	162 (55.1%)	
Primary CR				
	adjuvant chemotherapy	174 (95.1%)	79 (91.9%)	*p* = 0.407
NACT				
	interval debulking surgery	72 (41.9%)	96 (59.3%)	*p* = 0.001
Cytoreductive status				
	PDS			*p* = 0.014
	R0	138 (75.4%)	56 (65.1%)	
	R1	40 (21.9%)	17 (19.8%)	
	R2	9 (10.5%)	9 (10.5%)	
	Unknown	1 (0.5%)	4 (4.7%)	
	IDS			*p* = 0.897
	R0	60 (84.5%)	79 (83.2%)	
	R1	8 (11.3%)	10 (10.5%)	
	R2	3 (4.2%)	6 (6.3%)	
	Unknown	1 (1.4%)	1 (1.0%)	
1-year survival		293 (72.2%)	203 (69.0%)	*p* = 0.370
3-years survival		183 (45.1%)	106 (36.1%)	*p* = 0.017

## Data Availability

The original contributions presented in this study are included in the article. Further inquiries can be directed to the corresponding author.
